# A CBCT based cross sectional study on the prevalence and anatomical feature of C shaped molar among Jordanian

**DOI:** 10.1038/s41598-022-20921-1

**Published:** 2022-10-13

**Authors:** Taher Al Omari, Mustafa AlKhader, Ayfer Atav Ateş, Dian Agustin Wahjuningrum, Alaa Dkmak, Waheeb Khaled, Hazem Alzenate

**Affiliations:** 1 Department of Conservative Dentistry, Faculty of Dentistry, Jordan University of Science and Technology, Irbid, Jordan; 2 Department of Oral Medicine and Oral Surgery, Faculty of Dentistry, Jordan University of Science and Technology, Irbid, Jordan; 3 Department of Endodontics, Faculty of Dentistry, Istinye University, Istanbul, Turkey; 4 Department of Conservative Dentistry, Faculty of Dental Medicine, Universitas Airlangga, Surabaya, Indonesia

**Keywords:** Anatomy, Medical research

## Abstract

The prevalence and anatomical features of C-Shaped Mandibular Second Molars (MSMs) are rarely studied in Jordanian sub-population. This study then took a part to evaluate the prevalence of C-shaped in MSMs using cone-beam computed tomography (CBCT) in the Jordanian sub-population. It used a cross-sectional design and three thousand scans collected over eight years between 2011 and 2019. The data were then reviewed for whether they were fully formed of MSMs. A total of 2037 cases that had 2845 MSMs were evaluated to identify C-shaped canals at coronal, middle, and apical sites. An oblique slicing module perpendicular to the long axis of MSMs was used to evaluate the teeth. The type and frequency of C-shaped canals, as well as the correlations between sex and side (right/left) and between sex and groove direction (buccal/lingual) were measured using the chi-square test on SPSS software at the significance level of 95%. A total of 342 teeth of 243 patients were C-shaped molars, which comprised 12% of the patient’s teeth and 99 of them as a bilateral C-shaped canal with mean age of 40 years and sex ratio of 2:1 between female and male. With the limitations of this study, the lingual groove and type 3 were the most common properties of MSM. Besides, the Jordanian population mostly had C-shaped canals.

## Introduction

Anatomical variation in teeth is a natural phenomenon in human teeth. The failure of Hertwig’s epithelial root sheath fusion in lower molars may cause an uncommon anatomical variation, a thin ribbon-shaped canal^1^. Cooke and Cox were the first researchers who reported the C-shaped configuration in endodontic literature^2^. The occurrence of this variation differs among different populations; it can be lower than 5% in certain populations^3,4^ and as high as 40% in the Chinese population^5^.

This anatomical variations in human teeth between different populations are worth investigating; however, detecting the variations on a conventional radiograph requires advanced-imaging studies. Moreover, the presence of radiographic-fused roots on the conventional x-rays and panoramic reconstruction increases the prevalence of C-shaped molars by a factor of 17.2 times^6^. Many methods have been used to study the root canal morphology, such as the clearing technique, vulcanite casts duplicated, tooth sectioning, and microscopic evaluation, as well as radiographic micro–computed tomographic imaging and cone-beam computed tomography (CBCT)^7–9^. Many of the morphological studies are conducted using non-invasive methods such as CT and CBCT^5–8^. CBCT has its own advantages and disadvantages. Some of its advantages are that it is widely used and available to modify the visual field, the high-resolution images and the high confidence level^10^. Despite the advantages, most studies using the CBCT method have relatively a small sample size. To date, the prevalence of C-shaped lower molars in the Jordanian subpopulation was reported to be 10%^11^.

None of literature evaluates the prevalence of the C-Shaped mandibular second molars (MSM) in the Jordanian sub-population using the CBCT method. Therefore, this cross-sectional study aimed to verify the prevalence and anatomical features of C-shaped mandibular second molars in this population using CBCT imaging technology.

## Methods

### Study design

In this cross-sectional retrospective study, a pool of 3,000 CBCT images collected from north and middle territories of Jordan were initially evaluated for whether they were fully formed of mandibular second molars (MSMs). The images were collected over 8 years (2011–2019) at the Dental Teaching Hospital of Jordan University of Science and Technology and CBCT 3D Dental Imaging Center/Amman. This work has been reported in line with previous research using STROBE guidelines^12^ and investigating preferred reporting items for epidemiologic cross-sectional studies on root and root canal anatomy using cone-beam computed tomographic technology.^13^ The study procedures started from preparing, data analysis, and writing of this study. As the present study did not interfere with the psychological or physical of patient. This study was approved, and the informed consent is waived by Ethical Clearance Commission of the Institutional Review Board of Jordan University of Science and Technology (6/140/2021).

### Study population

Patients aged between 18 and 65 years were included in the analysis. Images were taken for various reasons not limited to endodontic purpose. Teeth with artefacts, root canal treatment, posts and core, crowns, pathologies affecting the root and impossibility to determine the tooth numbering were omitted from the study. KODAK 8100 and 9500 Cone Beam 3D System (Carestream, Rochester, NY) machines with a flat panel detector were used. CBCT scanning parameters for the 8100 CBCT machine were 0.15-mm as the voxel size, 81 kV as a tube voltage, 6.3 mA as the tube current and 15 s as the exposure time, representing two third of the sample size. Parameters for the 9500 CBCT machine were 0.2-mm as the voxel size, 90 kV as the tube voltage, 10 mA as the tube current and 10.8 s as the exposure time. Patients were asked to stop swallowing and to keep their teeth in maximum intercuspation. The occlusal plane was parallel to the floor, and the field of view was 5 × 5 cm and 15 × 9 cm, respectively.

#### Measurements and CBCT-based 3D examination on Kodak Dental Imaging Software

The CBCT sample images were collected over eight years from 2011 until 2019. After one-hour training conducted by a maxilla-facial radiologist with 12 years of experience, three graduate dentists, internally calibrated, were responsible for generating and simultaneously evaluating three cross-sectional CBCT slices perpendicular to the long axis for all MSMs. Their primary task was to evaluate the presence of C-shaped mandibular second molars. After the first selection of teeth, to remove any source of bias, the experienced radiologist and calibrated endodontist evaluated all cases in a darkened room. Kappa test was used for intrarater reliability. The interobserver reliability was high for all evaluated teeth related to C-shaped canals identification (Cohen’s Kappa of > 0.91) and classification (100% of agreement).

**Figure 1:**
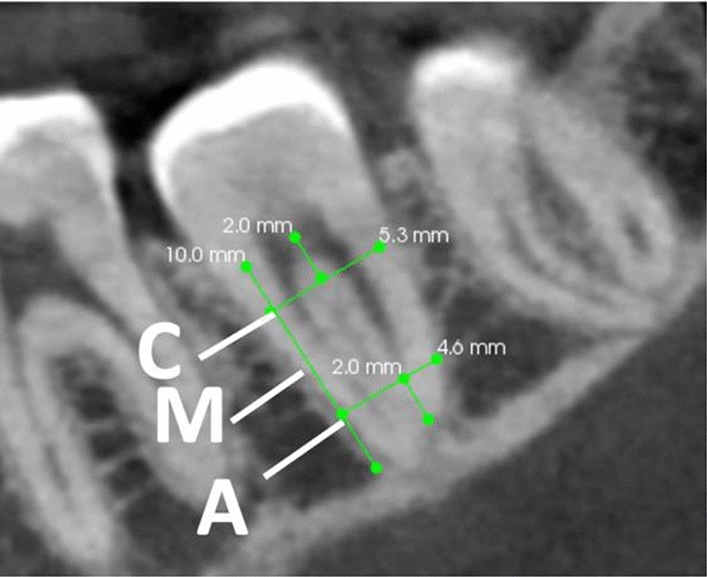
Representative sagittal CBCT view of a mandibular second molar generated by oblique slicing module. Exemplifying the three axial levels i.e., coronal (C), middle (M), and apical (A) at which the evaluation was performed.

The observers evaluated C-shaped canal scans following the examples from Alfawaz et al.^14^. The generated slices were coronal, middle and apical; coronal sections were taken 2 mm beneath the pulpal floor, where the middle floor was calculated by dividing the full length of the root by 2, and apical was measured 2 mm above the radiographic apex (Fig. 1).

To generate the cross-sectional slices, the oblique slicing module of the CS 3D imaging viewer version 3.10.4.0. was used. The sagittal oblique image views were created by moving the green bar at MSM over the horizontal section, and then the yellow bar was moved up to 90 degrees to the long axis of each MSM and vertically to the three previously determined sections (Fig. 1).

Shemesh et al.^3^ mentioned some following classifications, modified from Fan et al.^15^ to evaluate the CBCT images. Within the same framework, this current study classified C-shaped canals as three cross-sectional slices.

**Figure 2:**
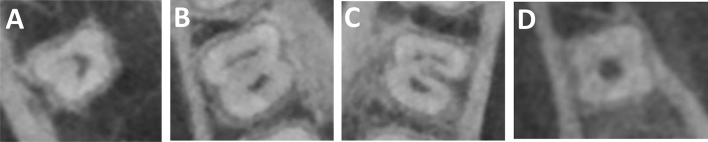
Example of oblique slicing module perpendicular to the long axis of a mandibular second molar. (**A**) Type 1—Continuous C with no separation or division, (**B**) Type 2—kidney-shaped semi-column, (**C**) Type 3—separate canals either two or three, (**D**) Type 4—one funnel-shaped large canal. (*B* buccal, *L* Lingual).

1. Type 1: continuous C with no separation or division (Fig. 2a)
2. Type 2: kidney-shaped semi-column (Fig. 2b)
3. Type 3: separate canals either two or three (Fig. 2c)
4. Type 4: one funnel-shaped large canal (Fig. 2d).

The groove (developmental depression in root surface) for each C-shaped root was also classified as buccal or lingual.

An LCD monitor with the installed dedicated CS 3D imaging viewer was set along with the screen settings adjusted to optimize the images for evaluation.

The chi-square test was used to measure the frequency and types of the C-shaped canals, the correlations between sex and side (right or left) and between sex and groove direction (buccal or lingual). IBM SPSS Statistics software (version 20.0; IBMCorp., Armonk, NY, USA) was used to proceed the data analysis with a significance level of 0.05.

### Ethics declaration

This present study was approved by the Institutional Ethics Committee of Jordan University of Science and Technology and was conducted according to the Guidelines of the Declaration of Helsinki.

## Results

### Patients’ characteristics

**Figure 3:**
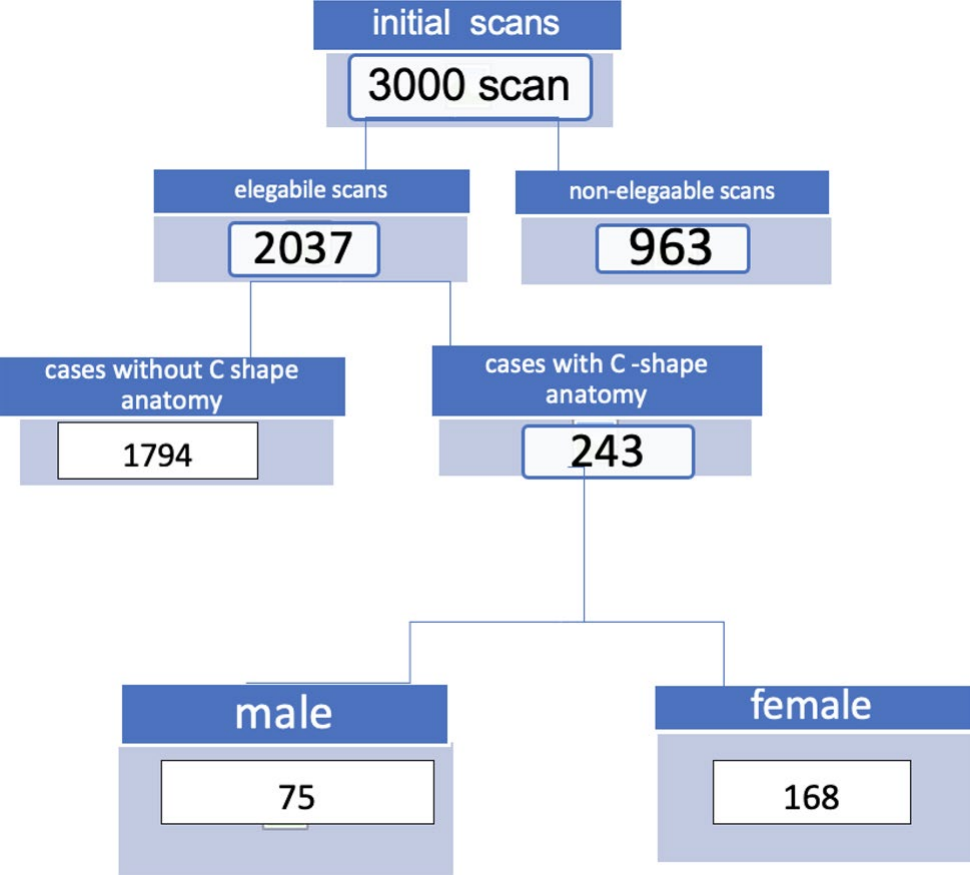
Flow diagram for the eligible cases.

Out of 3000 patients, a total of 2037 patients had fully formed MSMs, and 243 patients had C-shaped molars. The selected patients consisted of 75 males and 168 females with a mean age of 40 years. The total number of C-shaped molars was 342 teeth out of 2845 screened teeth. The flow diagram shown in Fig. 3. The prevalence of C-shaped canals in MSMs was 12.0%, while 99 patients had bilateral C-shaped canals.

No statistically significant differences were detected for sex (p > 0.05) and side of occurrence (left vs right side) (p > 0.05) on the formulation of C-shaped canals; however, statistically significant differences were detected between sex and groove direction (p < 0.05). Table 1 shows the analysis of the gender, side, and groove distribution of C-shaped canals in the population. Table 2 shows the analysis of the relationship between gender, side, and groove. Additionally, Table 3 indicates the classifications of C-shaped mandibular second molars modified from Fan’s classifications.

**Table 1:** Distribution analysis of gender, side, and groove distribution of C-shaped canals in mandibular molars of the Jordanian population.

	Number of included cases	C-shaped canal (%)
**Gender**		
Female	342	237 (69.3%)
Male	342	105 (30.7%)
**Side**		
Right	342	183 (53.5%)
Left	342	159 (46.5%)
**Groove**		
Buccal	342	72 (21.1%)
Lingual	342	270 (78.9%)

**Table 2:** Analysis of the relationship between gender, side, and groove.

	Female (%)	Male (%)	Total (%)
**Side**			
Right	126 (36.8%)	57 (16.7%)	183 (53.5%)
Left	111 (32.5%)	48 (14%)	159 (46.5%)
**Groove**			
Buccal	59 (17%)	13 (4%)	72 (21%)
Lingual	178 (52%)	92 (27%)	270 (79%)
Total	237	105	342

**Table 3:** Classifications of C-shaped mandibular second molars based on the modified Fan’s classifications.

	Type 1 (%)	Type 2 (%)	Type 3 (%)	Type 4 (%)	Total
Coronal third	100 (29.2%)	56 (16.4%)	121 (35.4%)	65 (19.0%)	342
Middle third	84 (24.6%)	58 (17.0)	138 (40.4%)	62 (18.1%)	342
Apical third	43 (12.6%)	23 (6.7%)	104 (30.4%)	172 (50.3%)	342

## Discussion

In this current study, the prevalence and configuration of C-shaped canals in MSMs were evaluated in different parts (coronal, middle, apical) of the teeth. The results of this study showed that the percentage of C-shaped canals in MSMs was 12% in the Jordanian subpopulation.

The canal configuration may vary according to the ethnic population (Table 4). Previous studies showed that the percentage of C-shaped canals in the second mandibular molar teeth ranged from 2.7% in the American population^2^ to 44.5% in the Korean population^16^. The pooled proportion of C-shaped mandibular second molars in East Asian countries (39.6%; 36.0–43.1%) was significantly higher than in other regions^13^. The prevalence of C-shaped canals in the Brazilian population was 15.3%, but it did not differ by gender or age. There was a significant prevalence of C-shaped canals in the mandibular second molars of the population studied^17^. Wide variations in the root and canal morphology of mandibular second molars were found in Emirates population with a relatively high prevalence of C-shaped canal configuration (17.9%)^18^. A more recent study showed the prevalence of C-shaped canal configuration in Iraqi sub-population was 17.4% which is similar to Emirates population and more than Jordanian sub-population. While the female/male ratio was similar to the current study. In Iraqi sub-population, a higher prevalence was found in women than men (10.4%)^19^. In both studies, women exhibited more C-shaped mandibular molars than men. This result is similar to a global study conducted by von Zuben et al.^20^.

**Table 4:** Incidence of C-shaped root canal configuration mentioned by in vivo studies.

Investigators	Race	Overall prevalence of C-shaped canals in mandibular first molar (%)	Overall prevalence of C-shaped canals in mandibular second molar (%)
Shemesh et al.^3^	Israel	0.16	4.6
Silva et al.^4^	Brazilian	1.7	3.5
Zheng et al.^5^	Chinese		39
Nejaim et al.^6^	Brazilian	2.39	14.32
Pan et al.^9^	Malaysian		48.7
Jin et al.^13^	Korean	–	44.5
Wang et al.^14^	Chinese	–	41.27
Ladeira et al.^15^	Brazilian	–	15.3
Khawaja et al.^16^	Emirati		17.9
Zhang et al.^31^	Chinese	29	29
Pawar et al.^32^	Indian	–	13.2
Alfawaz et al.^38^	Saudi	0.19	9.1
Abdalrahman et al	Iraqi	–	17.4

The results of this study showed that the value or the Jordanian population is 12% which is between these percentages. This result is consistent with a study conducted by Al-Qudah and Awawdeh^11^ discovering a similar prevalence (10%) in the same sub-population and less prevalence than a study by Ladeira et al.^18^ where 15.3% were found in Brazilian population^21^.

Table 4 shows the prevalence of C-shaped molars in different countries.

Wang et al.^17^ showed that the identification with radiographic examination showed 34.64% of C-shaped canal systems in mandibular second molars, and that using clinical examination showed 39.18%. The amount of C-shaped root canals diagnosed by radiographic method was statistically different from that by clinical examination and the combined examination^21^. However, it is challenging to obtain enough knowledge about the cross-sectional morphology and the canal systems with two-dimensional radiographs^3^. While CBCT images had more superior accuracy when compared to periapical radiographs that involve anatomical difficulties such as second mesiobuccal canals in maxillary molars and C-shaped canals in mandibular molars^22–24^. CBCT imaging is a good way to understand the full anatomy of teeth and plan root canal treatment.

Previous studies^25–27^ showed that changing the tomographic slice angulation or inclination could affect the visibility of anatomical structures, for example in the mandibular canals. The visibility of the canals was better when the slice inclination was perpendicular to the canal. Some new software (e-Vol DX, CDT Software, Bauru, Brazil) has been developed to visualize the teeth anatomy using slice inclination perpendicular to the canal^28–31^. Bueno et al.^24^ used this technique in an observational study on the anatomy of various teeth (maxillary central, mandibular molars and premolar).

Similarly, we generated perpendicular CBCT sections to the canals of MSMs by using the oblique slicing module, which enhanced the detection of different anatomy. Using this method in this current study likely resulted in the slightly higher prevalence of C-shaped canals than other studies conducted to the same ethnic group^11^. The C-shaped canal in endodontics has not clearly been defined yet, and different classifications of the canals have been used in previous studies^3,15,32^. In this current study, the CBCT images were evaluated according to modification criteria used by Shemesh et al.^3^. Since we performed our study with CBCT method, to avoid the low image quality in the apical part (2 mm), we followed Shemesh et al. to exclude category 5 of the Fan et al.^15^ classifications^3^.

According to this current study, type 1 (29.2%) and 3 (35.4%) were the most common in the coronal part. In addition, type 3 (40.4%) was the most common in the middle third of the canal, and type 4 was the most noticeable in the apical part (50.3%). The results related to the coronal part are partially relevant to what Shemesh et al.^3^ and Fan et al.^15^ found. They reported that type 1 was the most frequent. On the other hand, a study by Seo and Park^33^ showed that the frequency of type 2 was higher than other types. In Iraqi sub-population, type 2 was the most common in contrary to Jordanian sub-population where type 3 was the most common^19^. This inconsistency of certain images between the studies is likely influenced by the ethnicity of the study population and the use of various study designs. However, it may be an advantage for Jordanian clinicians because shaping and cleaning the type 3 root canal system is easier than type 1 and 2^5^.

In this current study, both right and left mandibular second molars were examined. Ninety-nine of the 243 patients or 40.7% of the patients had bilateral C-shaped second mandibular molars; therefore, we should be aware of C-shaped canals when treating the lower second molars. According to our research results, gender did not influence the side occurrence of C-shaped canals. This finding is in accordance with the studies conducted by Zhang et al.^34^ and Pawar et al.^35^. However, this current study found gender did affect the groove direction. These anatomical features are important both in endodontics and periodontology. These grooves are difficult to clean, cause plaque to accumulate, and are periodontally important anatomical formations on the root surface^36^. From the perspective of endodontics, the groove is considered a danger zone due to their low dentin thickness which tends to perforate^37^. Clinicians should be mindful about the direction of this groove while performing root canal treatment. In our study, the lingual groove was frequently seen, and previous research by Fan et al.^15^ showed similar result. However, this finding is in contrast to research by Ladeira et al.^18^.

The use of CBCT instead of micro‐computed tomography (µCT) is one of the limitations of this study. Although µCT gives more information about the details of the morphology, this technology is used for extracted teeth^38,39^. While extracted teeth that are generally pathological cannot simulate the anatomical features of second molars in Jordanian population. Despite this limitation, this study holds more contribution to research compared to previous research which used a clearing method with extracted teeth^11^ of the Jordanian population. Thus, our study gives novel information about the same population.

The strength of this study is that it used a large sample size using CBCT images and conducted evaluation by both the endodontist and radiologist. These advantages allow more accurate assessment of C-shaped canals; however, this study has other limitations related to data standardization which are the use of CBCT images acquired for different purposes other than endodontics and the collection of samples from two regions of Jordan. Future prospective research could be designed to accurately evaluate the C-shaped molars by sampling CBCT images collected for endodontic purpose from different geographical areas with specific parameters for the Jordanian population. Comparing the assessment method used for this study to new assessment tools (e-Vol DX, CDT Software, Bauru, Brazil) is worth to investigate in future research.

## Conclusion

In conclusion, the Jordanian population mostly had the lingual groove and type 3 properties of MSM with C-shaped canals. The prevalence of this morphology was 12%. Therefore, the clinicians should take these features into account and should combine CBCT and periapical radiography before starting endodontic treatment to reduce complications. With the thoughtful considerations on the features, a successful endodontic treatment may be achieved. Finally, it should be noted that the use of the oblique slicing module enhances the visibility of different anatomical features related to the detected C-shaped canals.

## Data Availability

The data used in this article are shared upon request to the corresponding author.
